# Dataset on the measurement of power in the hybrid vertical axis wind turbine in natural wind

**DOI:** 10.1016/j.dib.2020.105922

**Published:** 2020-06-25

**Authors:** Micha Premkumar Thomai, Seralathan Sivamani, Hariram Venkatesan

**Affiliations:** a Department of Mechanical Engineering, Hindustan Institute of Technology and Science, Chennai 603103, India; b Department of Aeronautical Engineering, Hindustan Institute of Technology and Science, Chennai 603103, India

**Keywords:** Hybrid VAWT, Savonius, Bach, Helical Helical bach, Magnetic levitation, Power measurement, Natural wind

## Abstract

The testing and performance data of the hybrid vertical axis wind turbines with magnetic levitation analyzed for low rated wind speeds is presented here. All the data are obtained by carrying out the investigations under natural wind conditions at Wind Turbine Research Station (WTRS), Kayathar, Tamilnadu, India. Four different wind turbine configurations namely helical Savonius - simple Savonius hybrid in two stage, helical Savonius - helical Bach hybrid in two stage, helical Bach – simple Bach hybrid in two stage, and single stage helical Bach. All the raw data obtained are processed and presented to compare the parameters among the different vertical axis wind turbine configurations.

Specifications table

**Table:** 

Subject	Energy Engineering and Power Technology
Specific subject area	Power measurement of several design combinations of Vertical Axis wind turbine configuration.
Type of data	Table Graph
How data were acquired	Data measured through Anemometer, Power analyser using NI DAQ on the basis of one-minute average.
Data format	Raw Analyzed
Parameters for data collection	Data of Wind speed, Power are measured in natural wind condition using instrumentation as per IEC 61,400–12–1
Description of data collection	The data of first two set of turbines i.e. Hybrid of helical Savonius and simple Savonius in two stage & Hybrid of helical Savonius and helical Bach in two stage are collected during the period from 11 July 2018 to 28 September 2018 (Time stamp: 11–07–2018 15:40 to 28–09–2018 23:59). The total number of data collected is 112,461 data sets. Subsequently, instrumentation of the next set turbines i.e., simple Bach and helical Bach hybrid in two stage and complete single stage helical Bach is carried out during end of September 2018. The data is collected during the period from 29 September 2018 to 24 October 2018 (Time stamp: 29–09–2018 00:01 to 24–10–2018 09:00). The total number of data collected is 36,541 data sets. All the stored / acquired data are on the basis of one-minute average.
Data source location	Institution: Wind Turbine Research Station, National Institute of Wind Energy. City/Town/Region: Kayathar, Tirunelveli, Tamil Nadu Country: India Latitude and longitude for collected data: 8.947°N, 77.77°E
Data accessibility	With the article

## Value of the data

• Data set on the performance analysis of hybrid vertical axis wind turbine with magnetic levitation system at low wind speed regimes gives an intuition about the suitability of installing the wind turbine at ground level to extract low rated wind energy.
• Data provides information on installing series of several small vertical axis wind turbines on roof top of urban buildings to produce power rather than installing a single large high capacity wind turbine.
• This data set will be useful to benchmark and validate the mathematical model or results of numerical simulations.

## 1 Data description

**Fig. 1:**
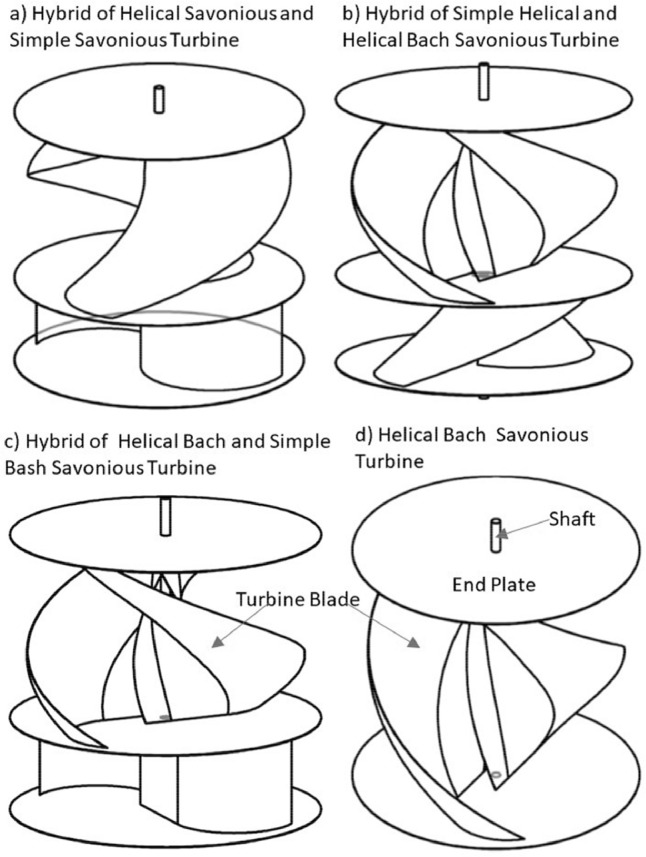
3D model of various types of turbine configurations a) Hybrid of Helical Savonius - simple Savonius in two stage (i.e., Wind turbine 1), b) Hybrid of Helical Savonius - Helical Bach in two stage (i.e., Wind turbine 2), c). Hybrid of Helical Bach - simple Bach in two stage (i.e., Wind turbine 3), d) Single stage helical Bach (i.e., Wind turbine 4).

**Fig. 2:**
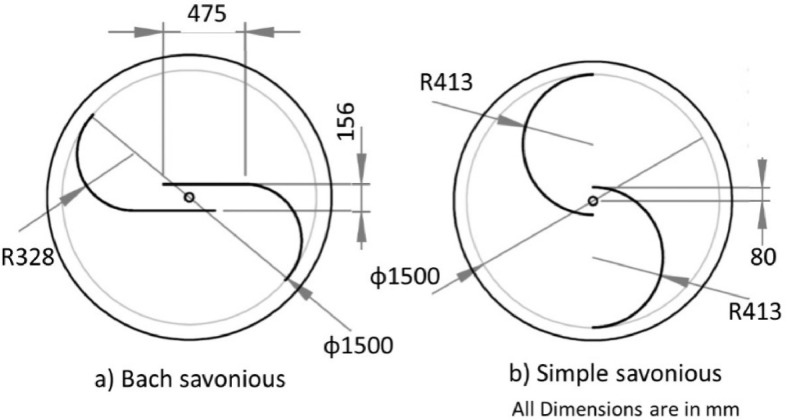
Dimensions of a) Bach Savonius b) Simple Savonius [12].

**Fig. 3:**
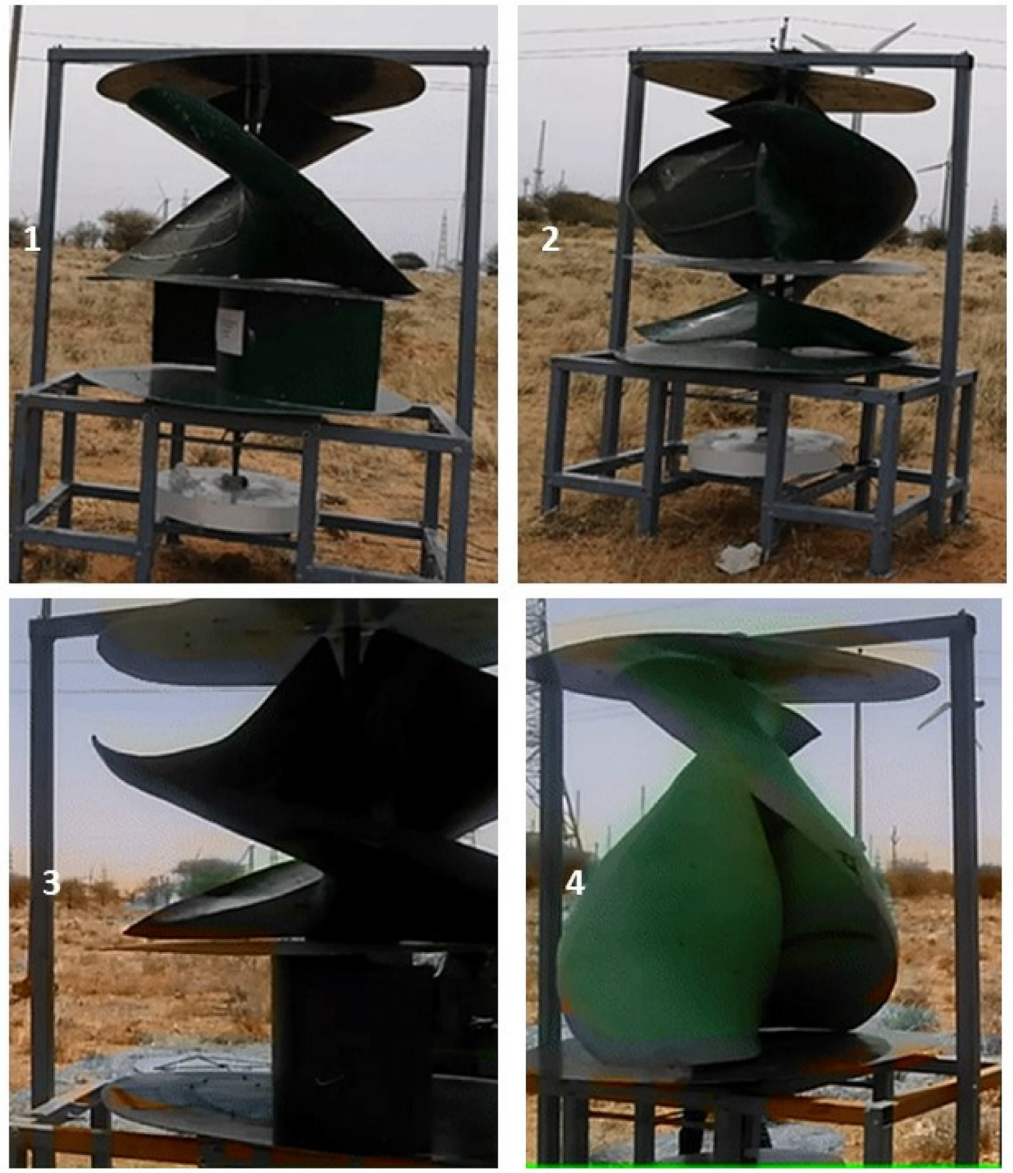
Four different vertical axis wind turbines tested at WTRS, Kayathar, 1. Hybrid of Helical Savonius - simple Savonius in two stage (i.e., Wind turbine 1), 2. Hybrid of Helical Savonius - Helical Bach in two stage (i.e., Wind turbine 2), 3. Hybrid of Helical Bach - simple Bach in two stage (i.e., Wind turbine 3), 4. Single stage helical Bach (i.e., Wind turbine 4).

The various vertical axis wind turbines fabricated, erected and tested at Wind Turbine Research Station (WTRS), Kayathar, Tamilnadu, India, are shown in Fig. 1. Four different wind turbines are tested in which three turbines are of two stage hybrid types and these are listed here. 1. Hybrid of helical Savonius and simple Savonius in two stage, 2. Hybrid of helical Savonius and helical Bach in two stage, 3. Hybrid of helical Bach and simple Bach in two stage, 4. Single stage helical Bach. Figs. 2 shows the detail dimensional data of Bach Savonious and Simple savonious vertical axis wind turbines tested for low wind speed regimes at natural wind conditions. Fig. 3 represents the actual picture of the wind turbines tested in WTRS. The various sensors used to measure the parameters are connected to the data logger. The measured parameters are listed in Table 1 along with the channel number, sensor type and its sampling frequency. The positions and locations of various sensors and instruments used during the test measurements are followed as per the international standard IEC 61,400–12–1. Table 2 gives the calibration coefficient of various data parameter measured in this work. Table 3 represents the database properties of the measured data. Table 4. Table 5, Table 6 and Table 7 illustrates the data for the power curve obtained from different vertical axis wind turbine configurations tested and its corresponding power curve are shown in Fig. 5, Fig. 7, Fig. 9, and Fig. 11 respectively. Fig. 12 shows the variation of power coefficient (C_p_) at different wind speeds for wind turbine 1 and wind turbine 2 respectively. Table 8 and Table 9 depict the data for the power coefficient of wind turbine 3 and wind turbine 4 respectively.

**Table 1:** List of measurement parameters.

Sl. No.	Measurement parameters	Channel number	Sensor type	Sampling frequency	Units
1	Wind Speed	A1	Analog	1Hz	m/s
2	Voltage	A3	Analog	1Hz	Watt
3	Power	A2	Analog	1Hz	KVAR
4	Power	A4	Analog	1Hz	Watts
5	Dump Load	C1	Counter	1Hz	Ohm

**Table 2:** Calibration coefficients.

Parameters	Total Calibration	
	Gain	Offset
Wind speed	18.750	−18.750
Voltage	30	0
Power	2500	−2500
Power	2500	−2500

**Table 3:** Database Properties.

Parameter	Database
Data sets up to maximum measured wind speed	112,461

**Table 4:** Power curve of hybrid of Helical Savonius - simple Savonius in two stage (i.e., Wind turbine 1) (on the basis of one minute average).

Wind speed(m/s)	P1(W)
0.00	0.00
1.87	6.42
2.27	17.70
2.78	14.10
3.28	9.28
3.77	14.56
4.27	15.02
4.77	16.01
5.26	21.40
5.76	29.36
6.25	40.29
6.75	52.95
7.24	66.84
7.74	80.31
8.23	95.78
8.73	113.27
9.22	124.70
9.74	140.61
10.22	153.01
10.72	171.03
11.23	183.33
11.75	207.28
12.21	222.36
12.69	181.59

**Table 5:** Power curve of hybrid hybrid of Helical Savonius - Helical Bach in two stage (i.e., Wind turbine 2), (on the basis of one minute average).

Wind speed (m/s)	P2 (W)
0.00	0.00
1.87	12.63
2.27	12.30
2.78	12.61
3.28	13.05
3.77	13.31
4.27	12.06
4.77	9.72
5.26	11.68
5.76	15.87
6.25	20.66
6.75	25.93
7.24	30.83
7.74	35.37
8.23	40.09
8.73	45.22
9.22	50.66
9.74	57.20
10.22	63.88
10.72	70.84
11.23	72.81
11.75	76.23
12.21	85.11
12.69	103.75

**Table 6:** Power curve hybrid of Helical Bach - simple Bach in two stage (i.e., Wind turbine 3) (on the basis of one minute average).

Wind speed (m/s)	P1 (W)
0.00	0.00
1.83	8.66
2.29	12.77
2.84	5.54
3.26	12.19
3.74	25.36
4.23	39.00
4.74	56.06
5.21	76.17
5.53	75.76

**Table 7:** Power curve of single stage helical Bach (i.e., Wind turbine 4) (on the basis of one minute average).

Wind speed(m/s)	P4(W)
0.00	0.00
1.83	13.37
2.29	13.67
2.84	13.87
3.26	14.63
3.74	14.64
4.23	14.76
4.74	14.39
5.21	14.38
5.53	14.30

**Table 8:** Power coefficient of hybrid of Helical Bach - simple Bach in two stage (i.e., Wind turbine 3).

Wind speed		
(m/s)	P1 (W)	C_p_
3.26	12.19	0.312751
3.74	25.36	0.430905
4.23	39	0.458027
4.74	56.06	0.467913
5.21	76.17	0.47876
Average C_p_	0.42

**Table 9:** Power coefficient of single stage helical Bach (i.e., Wind turbine 4).

Wind speed		
(m/s)	P4 (W)	C_p_
3.26	14.63	0.375352
3.74	14.64	0.248756
4.23	14.76	0.173346
4.74	14.39	0.120108
5.21	14.38	0.090384
Average C_p_	0.18	

## 2 Experimental design, materials and methods

To improve the performance of the turbine, various power augmentation techniques [1] like end plate [2], deflector plate [3], use of flaps [4], helix in the blade [5], multi-staging [6] are developed. The revolving motion of the wind turbine blade is primarily ascribable to the difference between the drag force acting on the advancing and returning blade. The geometry of the wind turbine influences the power production capability and geometric parameter named aspect ratio influence this aspect and it is directly proportional to it. Aspect ratio is defined as the ratio of height (H) to diameter of the turbine (D). There are so many literatures [7], [8], [9] that studied the effect of aspect ratio for the Savonius type wind turbine, and based on those studies it is observed that aspect ratio (A_s_) = *H*/D of 1 gives the better performance.

Height of the wind turbine, *H* = 1 m (fixed)

Aspect ratio $A_{s}=\frac{H}{D}$

1.0=1.0/D

Therefore, diameter of the wind turbine, D=1.0m

In addition to the aspect ratio (A_s_), another critical parameter that influences the performance of the wind turbine is overlap ratio (β). The overlap ratio is expressed as β = *e*/d, where e is the overlap distance and d is the chord length of the blade. The performance of the wind turbine is better for the overlap ratio (β) in the range between 0.10 and 0.25. Based on the literature, the overlap ratio (β) is kept as 0.15 [10]

The power coefficient (C_p_) of the vertical axis wind turbine (VAWT) can be improved by using end plates. It is observed that the diameter of end plate which is 10% more than the turbine diameter reported better performance [2]. As far as the number of blades is concerned, two bladed Savonius rotor gives the better performance [11]. Hence, all the data set reported here for different combinations of hybrid wind turbines consists of two blades in its configuration with end plates diameter measuring 1.10 times the diameter of turbine (refer Fig. 1).

Moreover, the double stage rotor is said to be superior to the corresponding single step turbine in terms of both torque and power characteristic. An attempt is made here to collect data from the two stage hybrid VAWTs having different combinations of helical and simple types, as shown in Fig. 1, to improve both the self starting characteristic and power features. Fig. 1 shows the 3D model of all the vertical axis wind turbines fabricated, erected and tested at Wind Turbine Research Station (WTRS), Kayathar, Tamilnadu, India. Four different wind turbines are tested in which three turbines are of two stage hybrid types and these are listed here.

1. Hybrid of helical Savonius and simple Savonius in two stage
2. Hybrid of helical Savonius and helical Bach in two stage
3. Hybrid of helical Bach and simple Bach in two stage
4. Single stage helical Bach

Altogether these hybrids are derived from the basic rotor dimensions of simple Savonius and Bach type Savonius as shown in Fig. 2 and these dimensions are arrived from the literature [1].

All the vertical axis wind turbines are fabricated using fibre reinforced plastic (FRP) material as it is found to be the best choice of the material for fabricating the turbine rotor, based on the criteria of less price, easy to build, low weight, good resistance to outside element, good rigidity, and ability to recycle.

The focus of this data set is to identify the design of magnetically levitated hybrid vertical axis wind turbine which develops high torque, high power along with better self starting ability. This data set is obtained based on the wind turbines developed as per the sanction order of the MNRE project and review recommendations of the project monitoring committee held on 11 July 2017. The system consists of four different configurations of vertical axis wind turbines coupled to generators with rated capacity of 300 Watts.

1. Hybrid of Helical Savonius - simple Savonius in two stage (i.e., Wind turbine 1)
2. Hybrid of Helical Savonius - helical Bach in two stage (i.e., Wind turbine 2)
3. Hybrid of Helical Bach - simple Bach in two stage (i.e., Wind turbine 3)
4. Single stage helical Bach (i.e., Wind turbine 4)

These wind turbines, as shown in Fig. 3, are erected and tested for low rated wind speeds at the Wind Turbine Research Station, Kayathar, Tamil Nadu.

Initially, instrumentation for the first two sets of turbines, i.e., simple Savonius and helical Savonius hybrid in two stage and helical Savonius and helical Bach hybrid in two stage, both coupled with generators of capacity 300 W is carried out during July 2018. The data is collected during the period from 11 July 2018 to 28 September 2018 (Time stamp: 11–07–2018 15:40 to 28–09–2018 23:59). The total number of data collected is 112,461 data sets.

Subsequently, instrumentation of the next set turbines i.e., simple Bach and helical Bach hybrid in two stage and complete single stage helical Bach is carried out during end of September 2018. The data is collected during the period from 29 September 2018 to 24 October 2018 (Time stamp: 29–09–2018 00:01 to 24–10–2018 09:00).The total number of data collected is 36,541 data sets.

The positions and locations of various sensors and instruments used during the test measurements are followed as per the international standard IEC 61,400–12–1 [13].

The measurement procedure is followed as described in IEC 61,400–12–1 standards [13]. All the stored / acquired data are on the basis of one-minute average.

The data selected for the power curve measurements are in accordance with IEC 61,400–12–1 standards [13] and it is filtered as per the condition named “Data eliminated due to integrity issues” in the IEC 61,400–12–1 standards. The data set reported here are on the basis of the measurements made during the period from 11July 2018 to 24 October 2018.

Based on the tests carried out at prevailing natural wind conditions, the scattered and binned power curves of various wind turbines are plotted and it is shown in Fig. 4–11. The data obtained for the power curves of various wind turbines are listed in Table 4 to 7.

**Fig. 4:**
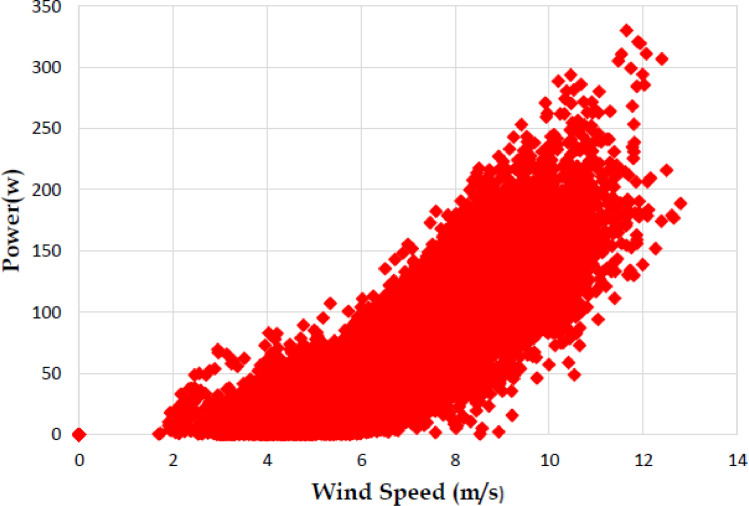
Scattered power curve of Hybrid of Helical Savonius - simple Savonius in two stage (i.e., Wind turbine 1) (on the basis of one minute average).

**Fig. 5:**
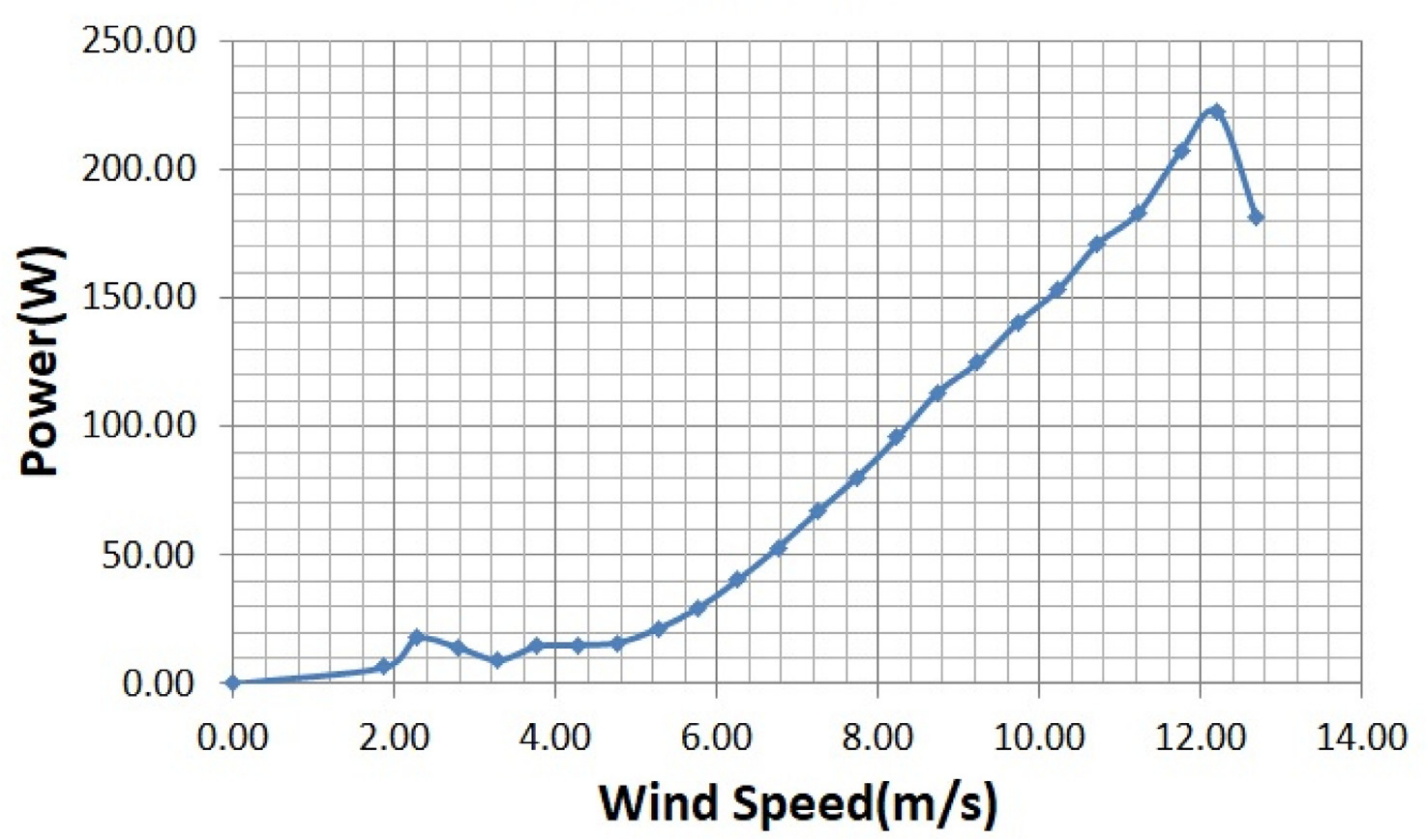
Binned power curve of Hybrid of Helical Savonius - simple Savonius in two stage (i.e., Wind turbine 1) (on the basis of one minute average).

**Fig. 6:**
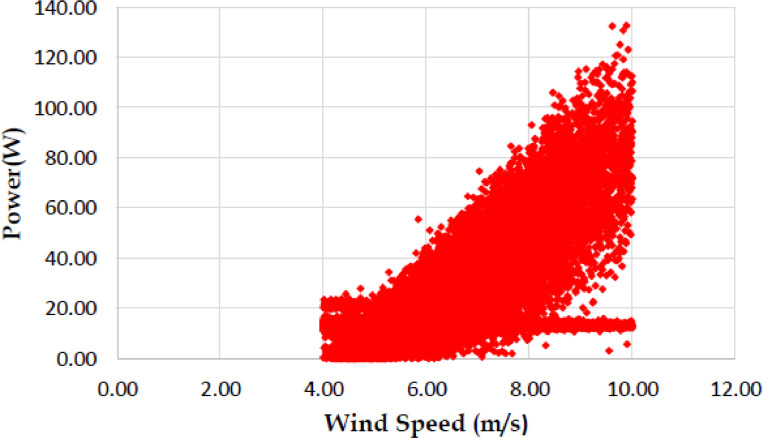
Scattered power curve of Hybrid of Helical Savonius - Helical Bach in two stage (i.e., Wind turbine 2), (on the basis of one minute average).

**Fig. 7:**
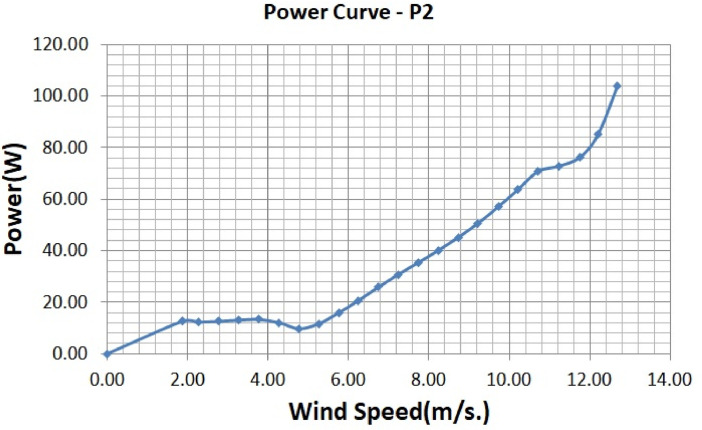
Binned power Curve of Hybrid of Helical Savonius - Helical Bach in two stage (i.e., Wind turbine 2), (on the basis of one minute average).

**Fig. 8:**
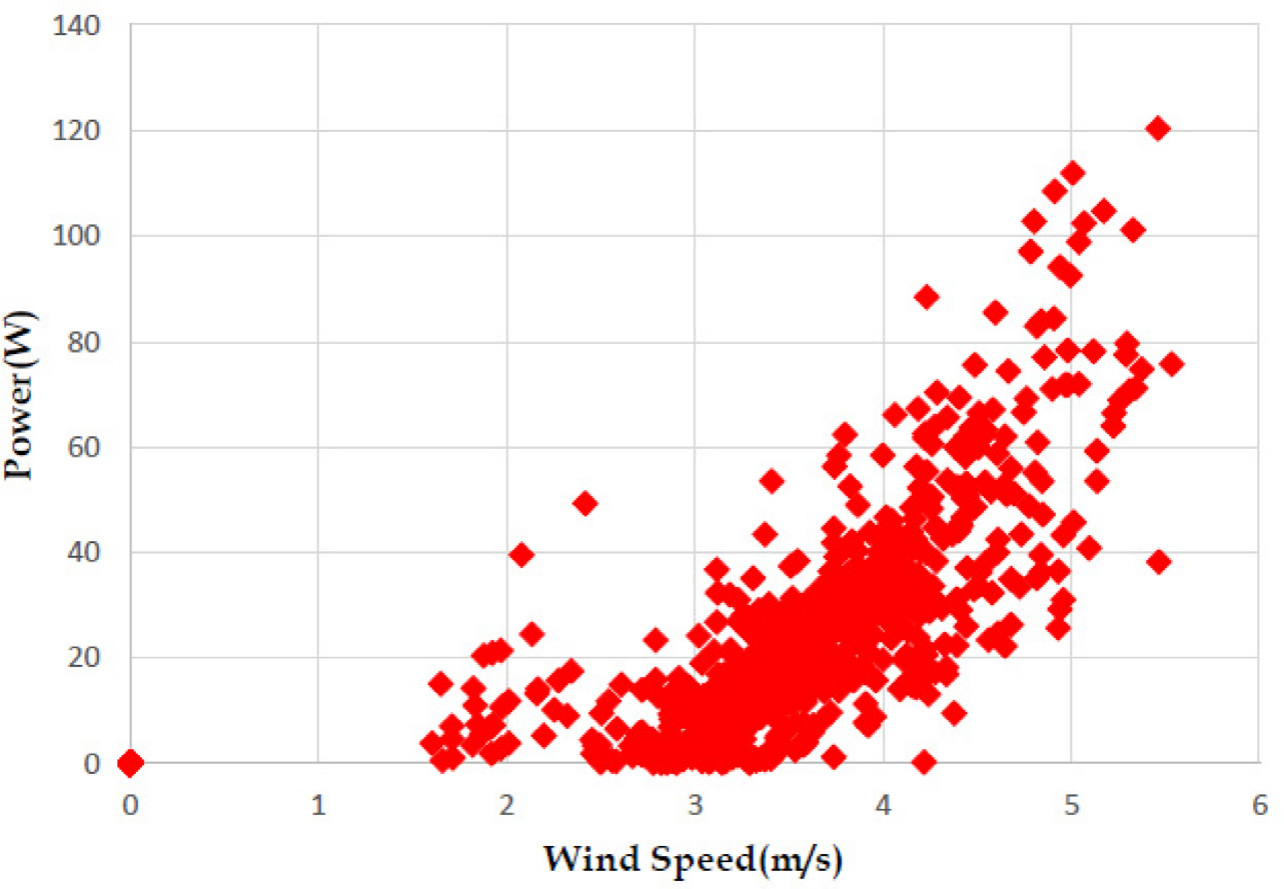
Scattered power curve of Hybrid of Helical Bach - simple Bach in two stage (i.e., Wind turbine 3) (on the basis of one minute average).

**Fig. 9:**
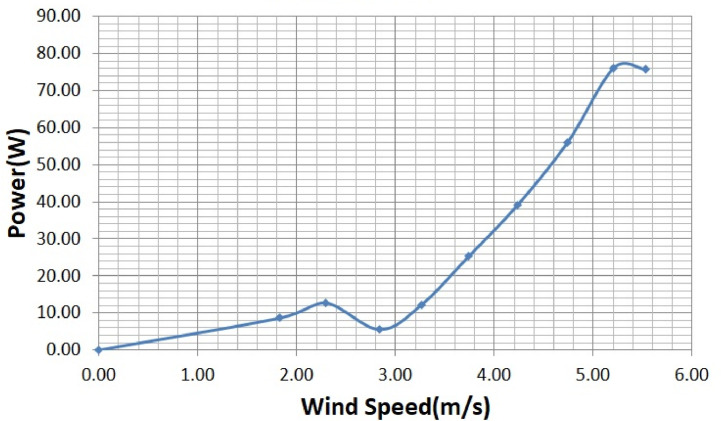
Binned power curve of Hybrid of Helical Bach - simple Bach in two stage (i.e., Wind turbine 3) (on the basis of one minute average).

**Fig. 10:**
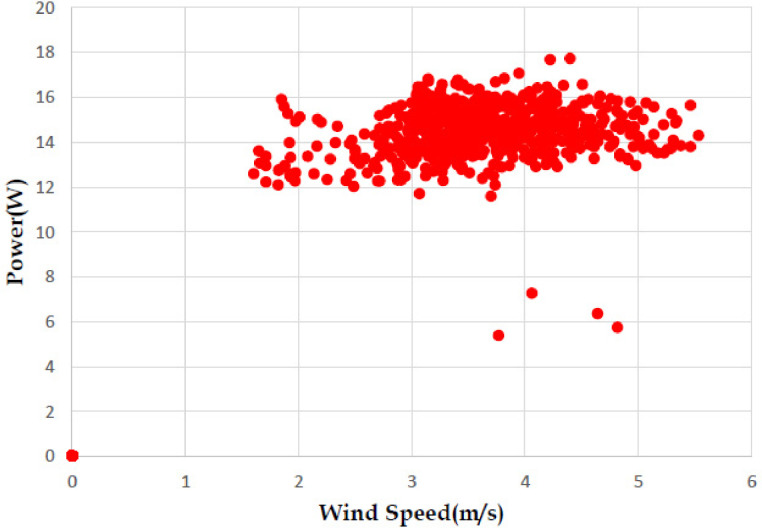
Scattered power curve of Single stage helical Bach (i.e., Wind turbine 4) (on the basis of one minute average).

**Fig. 11:**
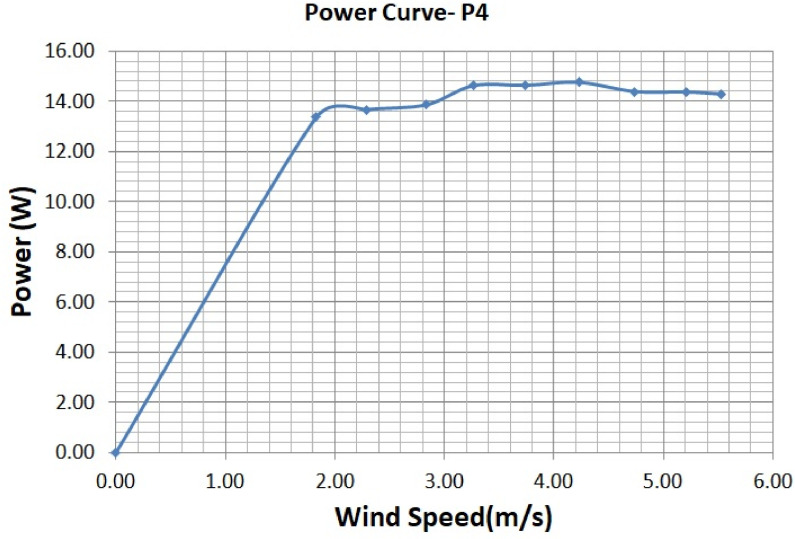
Binned power curve of Single stage helical Bach (i.e., Wind turbine 4) (on the basis of one minute average).

**Fig. 12:**
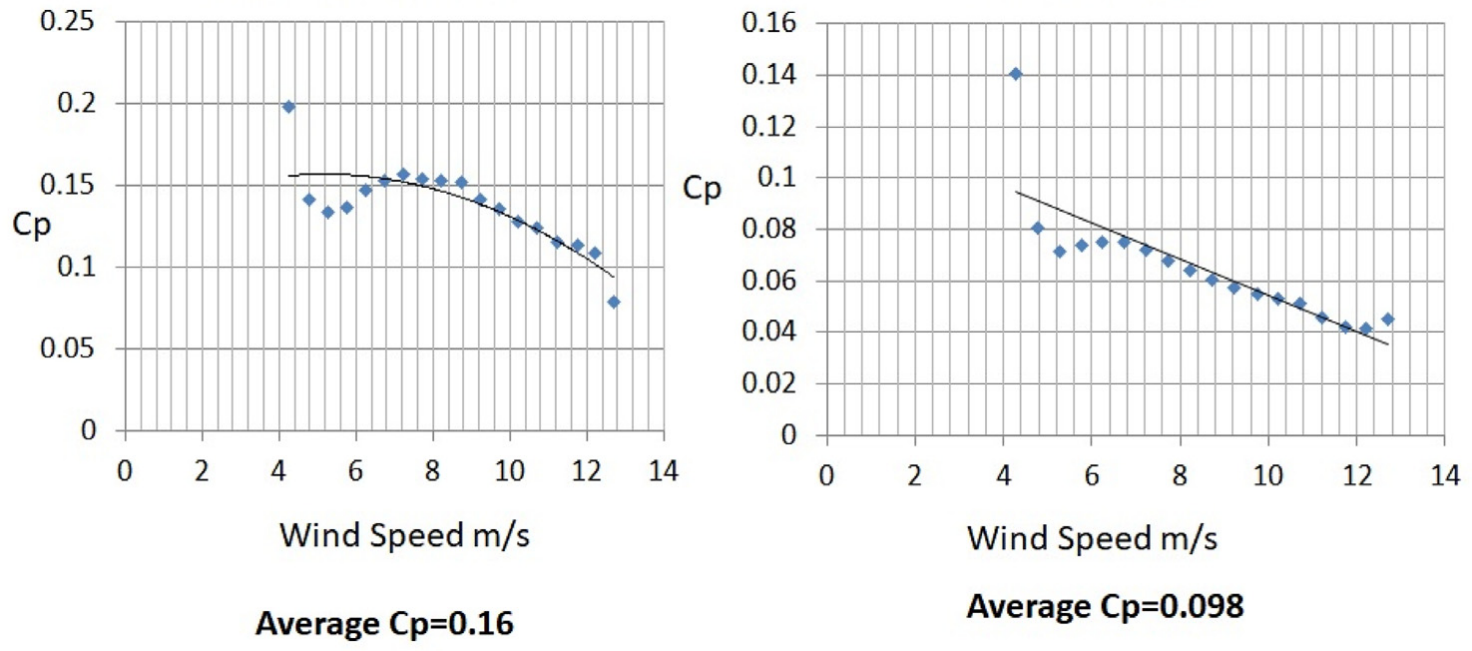
Variation of power coefficient for a) Hybrid of Helical Savonius - simple Savonius in two stage (i.e., Wind turbine 1) and b) Hybrid of Helical Savonius - Helical Bach in two stage (i.e., Wind turbine 2).

In order to ensure low wind speed testing conditions, all the vertical axis wind turbines are kept at the ground level with coordinates 8.947°N latitude and 77.774°E longitude in WTRS, Kayathar, Tamilnadu, India. As considerable portion of the energy are lost due to friction in the ball bearing arrangement, the focus in now towards zero frictional losses for small wind turbines. The concept of levitation caused by repulsion of opposite pole in the permanent magnet reduces the friction between the stator and wind turbine rotor thereby increasing the net power output of the wind turbine [14], [15]. Therefore, the passive permanent magnetic bearing (PPMB) is successfully designed, developed and configured in all the tested vertical axis wind turbine configurations.

The range and values of various parameters are as follows: number of buckets is two; nominal free stream velocity varies from 2 m/s to 14 m/s; Reynolds number per meter, 4.32 × 10^5^ and 8.67 × 10^5^; rotor height and diameter is 1.5 m and 1.5 m; bucket overlap is 0.10 m. The measured test variables are wind speed, voltage, power and dump load. The measurement procedure followed is as described in IEC 61,400–12–1 [13]. All stored / acquired data are on the basis of one-minute average. In order to compare the performance of different vertical axis wind turbine configurations, a non-dimension number named power coefficient (C_p_) is used. Based on the data listed and indicated through figures, it is observed that the average power coefficient (C_p_) of wind turbine 1 is 0.16, whereas the average Cp for the wind turbine 2 is 0.098. Moreover, the average power coefficient (C_p_) of wind turbine 3 is 0.42 and it is 0.18 for wind turbine 4. Based on the data set, it is observed that the power coefficient (C_p_) of two stage hybrid comprising simple Bach and helical Bach vertical axis wind turbine (Wind turbine 3) increased by nearly 2.6 times at low wind speed conditions compared to wind turbine 1. Based on the data available, it is found that Hybrid of Helical Bach - simple Bach in two stage (i.e., Wind turbine 3) is the suitable configuration for low rated wind regimes with good self starting.

## Declaration of Competing Interest

The authors declare that they have no known competing financial interests or personal relationships which have, or could be perceived to have, influenced the work reported in this article.
